# Supplementary material for: Detection of H5N1 High Pathogenicity Avian Influenza Viruses in Four Raptors and Two Geese in Japan in the Fall of 2022
Source: Viruses. 2023 Sep 1;15(9):1865. doi: 10.3390/v15091865 (PMC10537537; doi:10.3390/v15091865)
Supplement: Supplementary file 1 [file viruses-15-01865-s001.zip › viruses-2577946-supplementary.pdf]

Table S1. Northern Pintail Population in Yatsu-higata or Yatsu Tidal Flat, Chiba Prefecture, Japan.

		2020	2021	2022
September	Early	0	0	0
	Middle	0	0	0
	Late	0	0	3
October	Early	0	0	4
	Middle	0	31	73
	Late	5	0	102
November	Early	44	14	214
	Middle	16	7	188
	Late	4	305	3

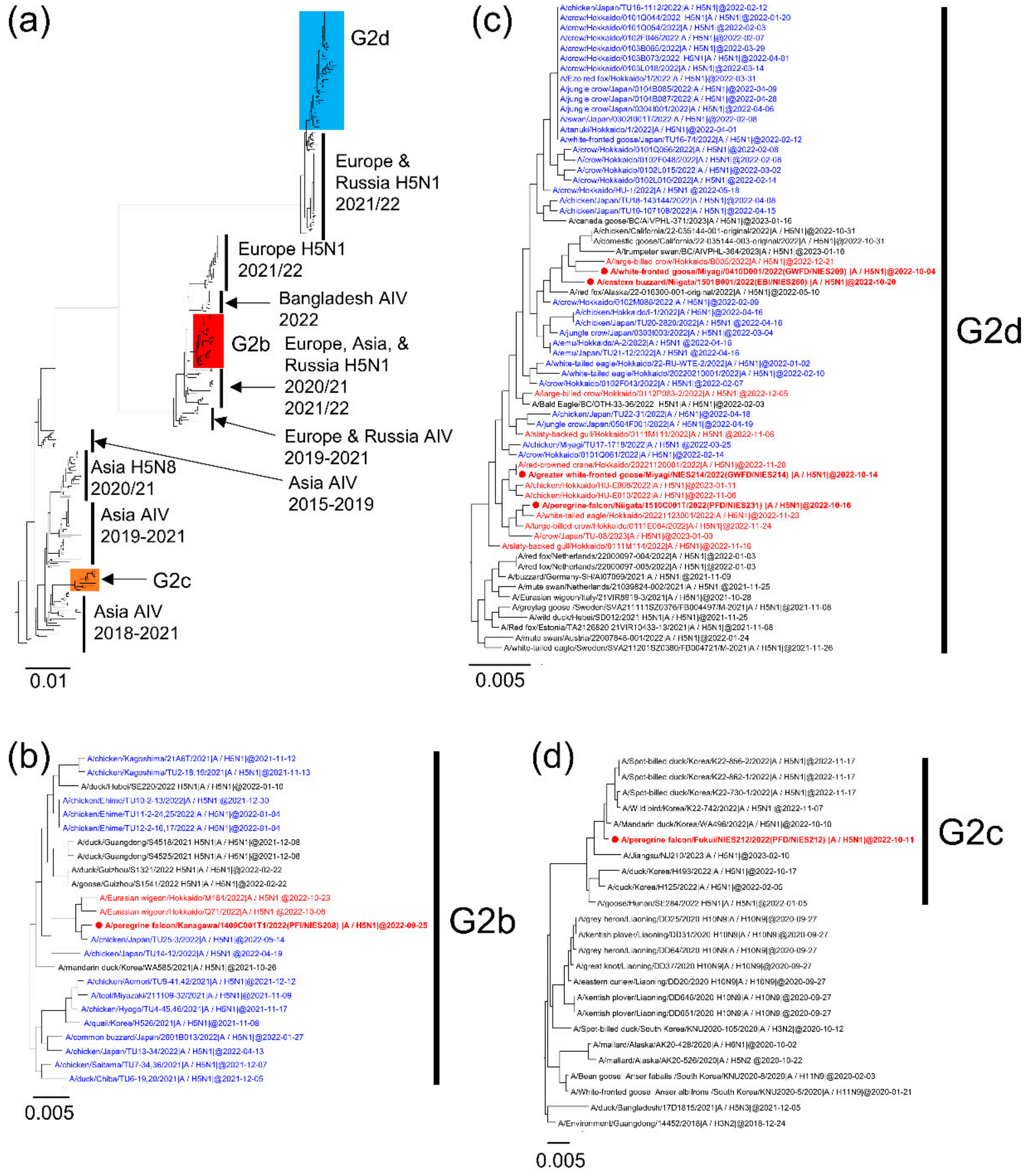

Figure S1. phylogenetic trees of the PB2 gene segment.

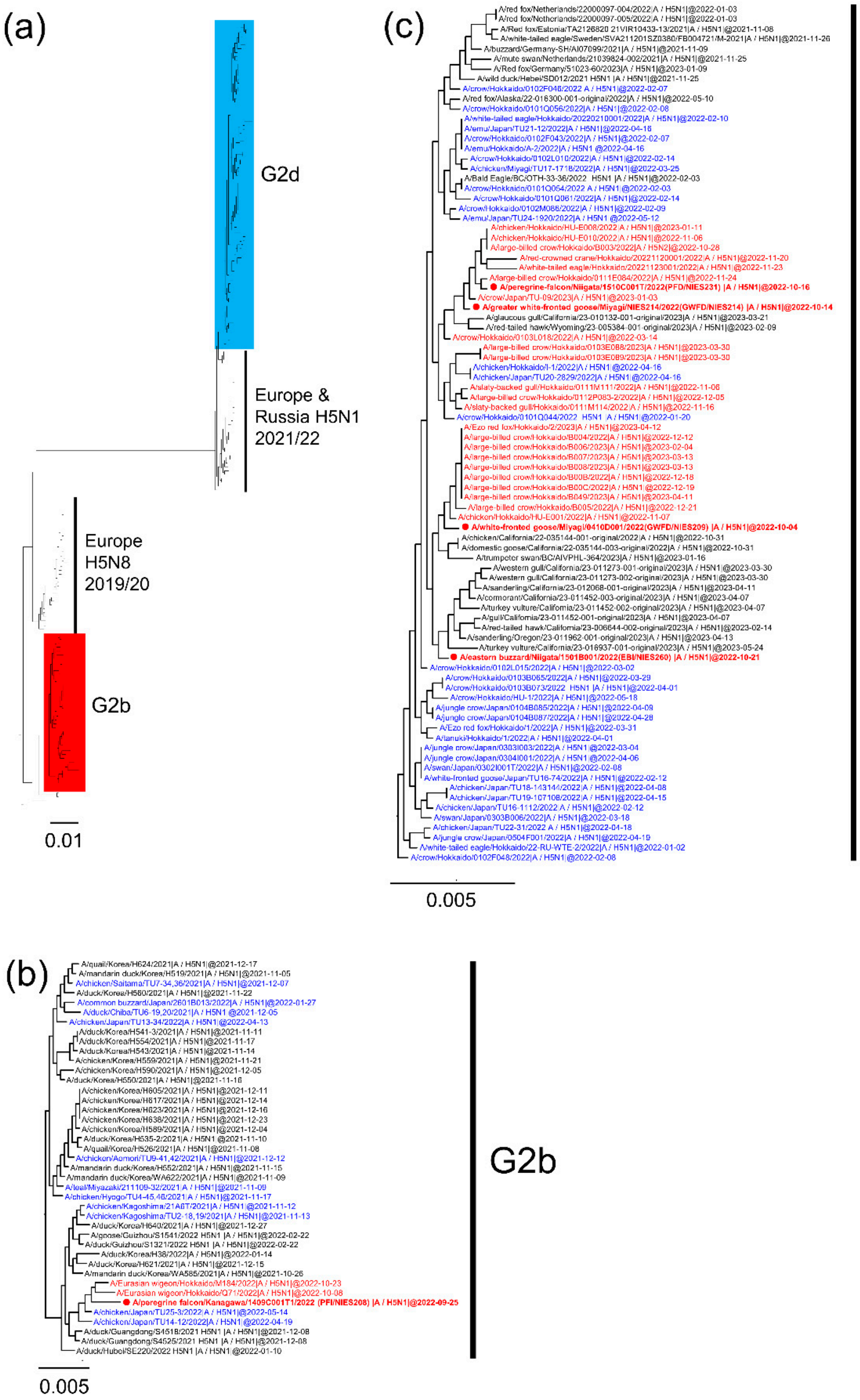

Figure S2. phylogenetic trees of the PB1 gene segment.

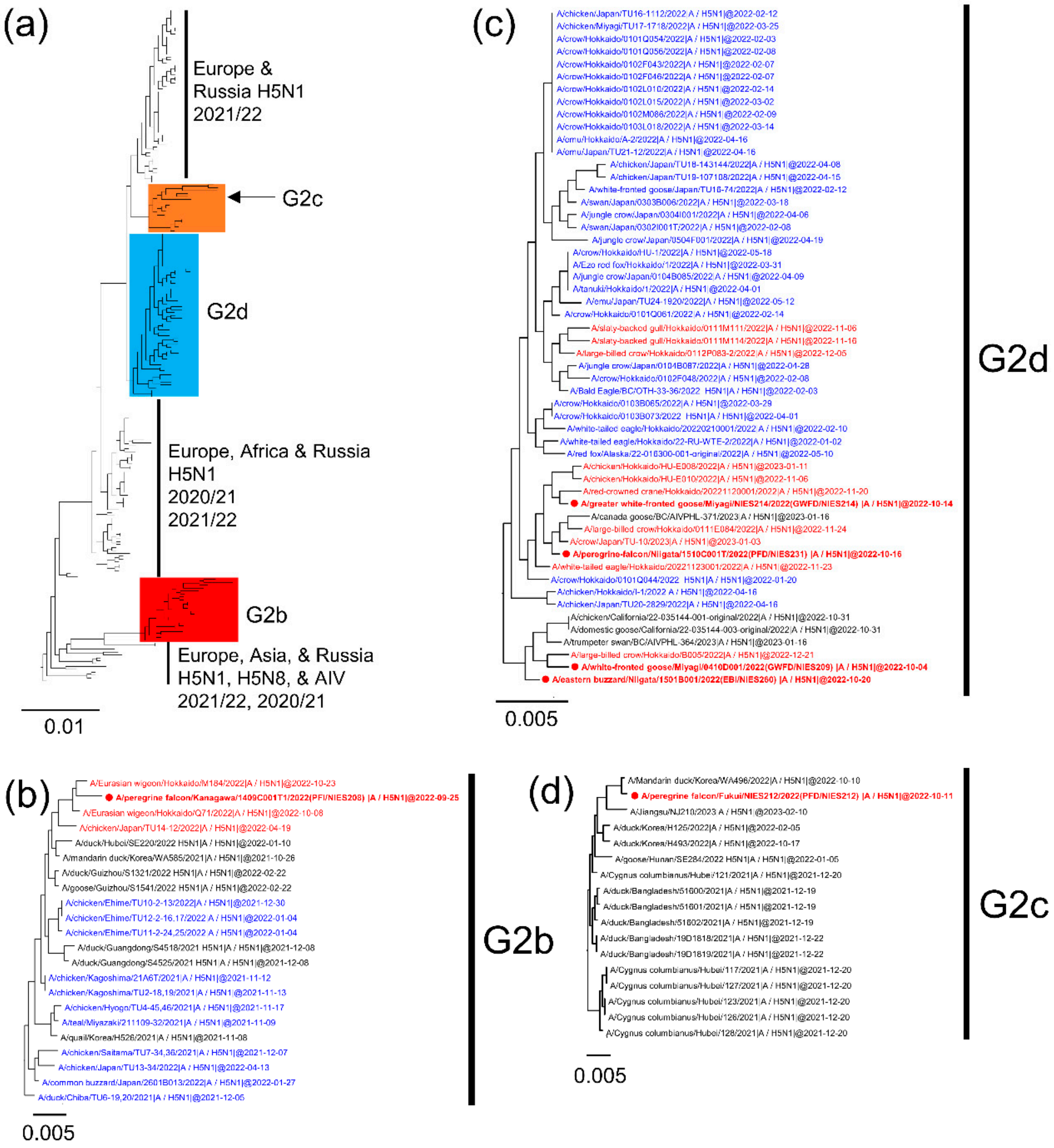

Figure S3. phylogenetic trees of the PA gene segment.

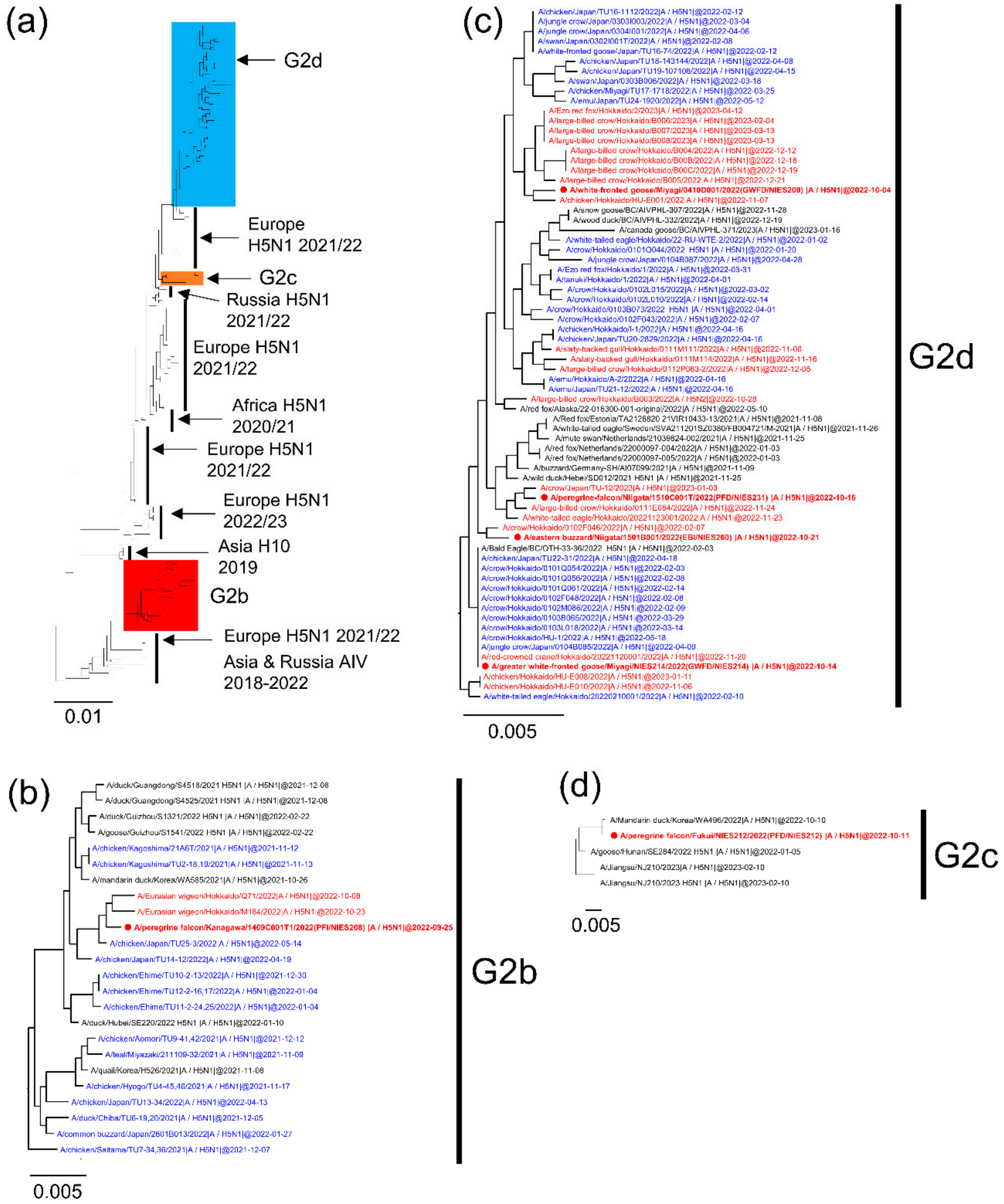

Figure S4. phylogenetic trees of the NP gene segment.

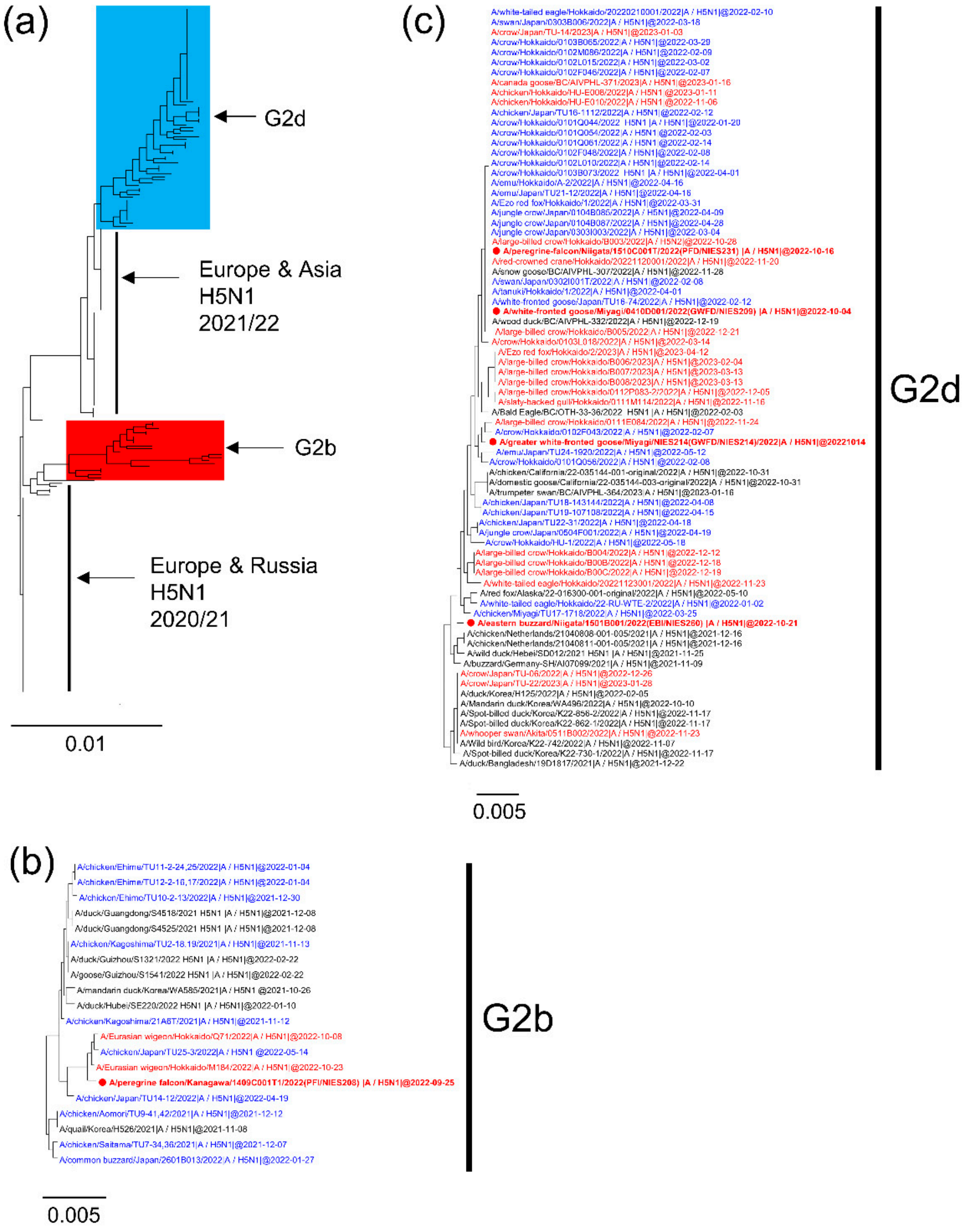

Figure S5. phylogenetic trees of the MP gene segment.

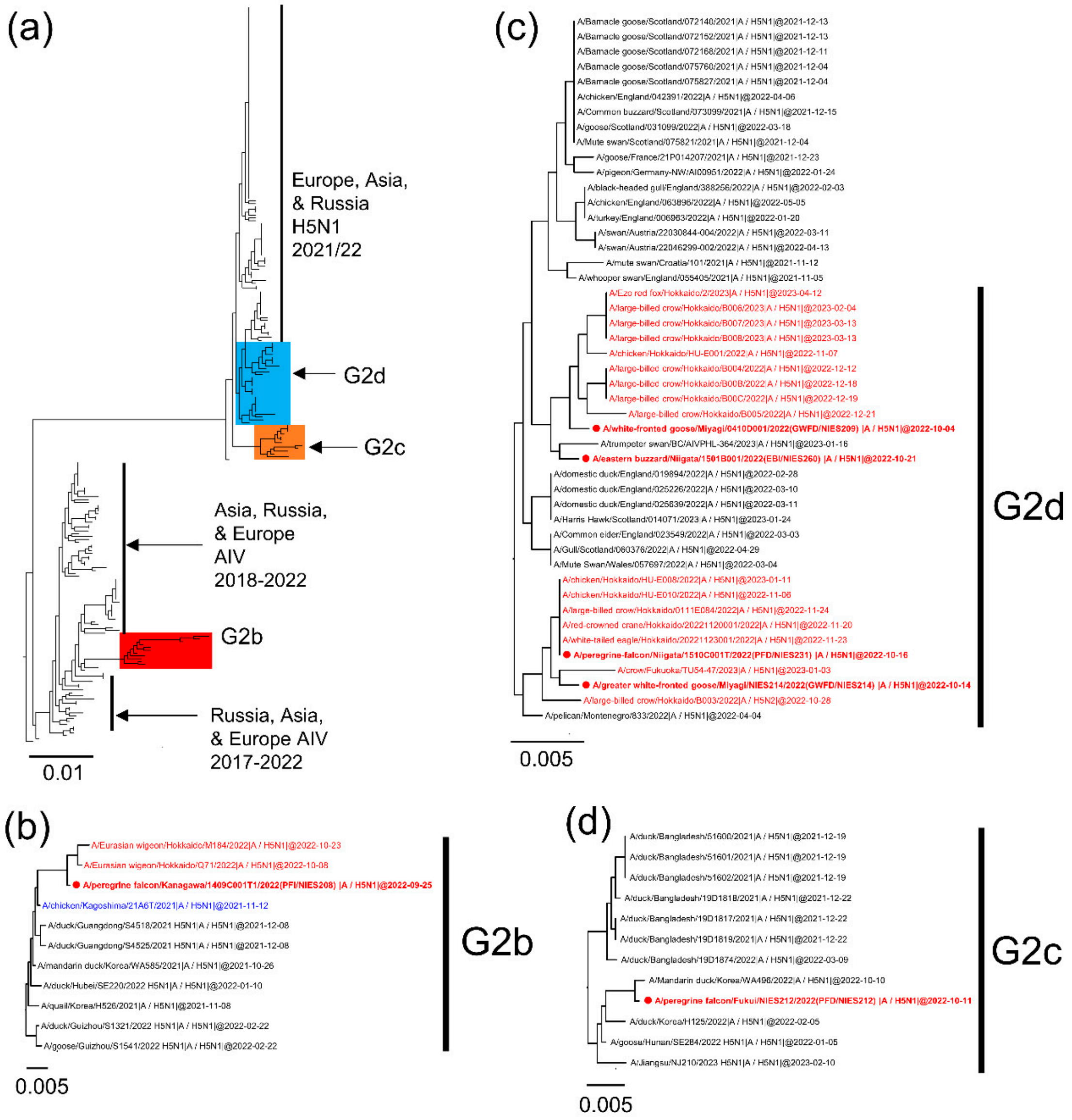

Figure S6. phylogenetic trees of the NS gene segment.
